# Mechanical and Metallurgical Evaluation of 3 Different Nickel-Titanium Rotary Instruments: An In Vitro and In Laboratory Study

**DOI:** 10.3390/bioengineering9050221

**Published:** 2022-05-20

**Authors:** Alessio Zanza, Paola Russo, Rodolfo Reda, Paola Di Matteo, Orlando Donfrancesco, Pietro Ausiello, Luca Testarelli

**Affiliations:** 1 Department of Oral and Maxillo-Facial Sciences, Sapienza University of Rome, Via Caserta 06, 00161 Rome, Italy; alessio.zanza@uniroma1.it (A.Z.); orlando.donfrancesco@uniroma1.it (O.D.); luca.testarelli@uniroma1.it (L.T.); 2 Department of Chemical Engineering, Materials, Environment DICMA, La Sapienza University of Rome, Via Scarpa, 00161 Rome, Italy; paola.russo@uniroma1.it (P.R.); p.dimatteo@uniroma1.it (P.D.M.); 3 Department of Anatomy, Histology, Forensic Medicine and Orthopedics, Sapienza University of Rome, 00161 Rome, Italy; 4 School of Dentistry, University of Naples Federico II, Via S. Pansini 5, 80131 Naples, Italy; pietausi@unina.it

**Keywords:** bending ability, cyclic fatigue, differential scanning calorimetry, endodontics, Nickel-titanium Rotary Instruments, scanning electron microscopy, torsional strength

## Abstract

An in-depth evaluation of the mechanical and metallurgical properties of NiTi instruments is fundamental to assess their performance and to compare recently introduced instrument with widespread ones. According to this, since there are no data on this topic, the aim of the study was to mechanically and metallurgically evaluate an instrument recently introduced into the market (ZenFlex (ZF)), by comparing it with two well-known instruments with similar characteristics: Vortex Blue (VB) and EdgeSequel Sapphire (EES). According to this, 195 instruments were selected: 65 ZF, 65 VB and 65 EES. Each group was divided in subgroups according to the mechanical tests (i.e., cyclic fatigue resistance, torsional resistance and bending ability; (*n* = 20)) and the metallurgical test (differential scanning calorimetry (*n* = 5)). A scanning electron microscopy was performed to verify the causes of fracture after mechanical tests (cyclic fatigue and torsional tests). According to results, VB showed the highest flexibility and cyclic fatigue resistance in comparison to the other instruments, with a statistically significant difference (*p* < 0.05). Regarding torsional resistance, EES showed the lowest value of torque at fracture, with a statistically significant difference, whilst the comparison between ZF and VB showed no statistically significant difference (*p* > 0.05). DSC analysis pointed out that VB had the highest austenite start and finish temperatures, followed by ESS and then ZF. ESS sample showed the highest martensite start and finish temperatures followed by VB and ZF. Considering the results, it can be concluded that VB showed the best mechanical performance during static tests in comparison to ESS and ZF. This is fundamentally due to the interaction of parameters such as instrument design and heat-treatments that are able to enhance its mechanical performance.

## 1. Introduction

Since the introduction of Nickel-Titanium (NiTi) alloy as the material of choice for the manufacturing of endodontic rotary instruments, the success rate of root canal treatments has increased incredibly [1,2,3]. The major limitations of manual instrumentation with stainless-steel files are fundamentally related to stiffness and low flexibility of files with great diameter, that could lead to iatrogenic errors such as zip, ledge, canal transportation or perforation [4,5]. Those drawbacks have been partially overcome with the NiTi alloy thanks to its metallurgical properties such as superelasticity, shape memory effect and possibility to ensure the use of endodontic instruments with an increased taper at higher speed without an excessive risk of fracture due to bending or cyclic fatigue, improving the quality of root canal shaping and therefore of root canal irrigation and filling [6]. 

In the last decades, those indisputable advantages have led to a greater use and production of NiTi endodontic systems, with a consequent increased interest of manufacturers and companies. In fact, in comparison to the early 2000s the number of endodontic NiTi companies increased, offering to clinicians a wide range of choice [7]. Moreover, recently the endodontic market has seen the birth of several companies which have begun an economic policy focused on the manufacturing of alternatives to the premium brand instruments as replica-like systems, with the aim to take a slice of the endodontic market thanks to the reduced sales prices [8,9]. Furthermore, the technology development keeps growing, so much so that manufactures continuously introduce into the market new instruments. According to this, it is crucial to evaluate the mechanical and metallurgical properties of recently introduced instruments in order to assess their mechanical limit and performance to give clinicians scientific validity of the products [10].

As thoroughly demonstrated, the two main causes of intracanal separation are the cyclic fatigue and the excessive torsional load or the combination of both [11,12,13,14]. The first one is an inevitable consequence of the rotation of the instrument inside a curve canal that generates tension and compression strain cycles in the region of maximum curvature. The second one is caused by the blockage of a part of the instruments, more frequently the tip, whilst its coronal part continues to rotate until the plastic limit of the alloy is exceeded and the fractur occurs [1]. The fracture resistance of NiTi rotary instruments is influenced by several factors, among which the instrument-related factors have a key role [15]. Those includes the morphology and design of the instruments such as diameter, pitch, helix angle, taper, rake angle, length, cross sectional design and polar moment of inertia, shaft length and heat treatment [7,15,16,17,18,19]. Regarding the latter, it is able to influence the transformation temperature range (TTR) and, thus, the crystallographic phase of the instrument at ambient or intracanal temperatures, giving the possibility to obtain martensitic instrument at ambient temperature. In fact, the NiTi alloy could be organized in two different forms: the austenite, characterized by a crystal structure with a body-centered cubic lattice stable at higher temperature and the martensite, characterized by a closely packed hexagonal lattice which guarantees more flexibility and cyclic fatigue resistance with enhanced plasticity [7]. Obviously, in order to have an in-depth comprehension of the mechanical properties of NiTi instruments it is imperative to know their metallurgy such as the points of the martensite start temperature (Ms) and the martensite finish temperature (Mf) within which an increasing amount of austenite is transformed in martensite, and the austenite start temperature (As) and austenite finish temperature (Af) within which an increasing amount of martensite is transformed in austenite. The most common analysis performed to assess the TTR and the effect of heat-treatments is the differential scanning calorimetry (DSC), in which the difference in thermal power supplied to a test specimen and an inert control specimen heated at the same rate is measured very accurately [20].

Recently, Kerr Corporation launched a new NiTi rotary system used in a continuous motion the ZenFlex (ZF; Kerr Corporation, Pomona, CA, USA) characterized by 1 mm maximum instrument diameter with the purpose to maintain more tooth structure after root canal treatment. Moreover, the manufacturer claimed of ensuring an increased cyclic fatigue and torsional resistance in comparison to other comparable instrument brands thanks to the proper heat-treatment and the innovative design of the ZenFlex (https://embed.widencdn.net/download/kavokerr/zq1mgdwywj/ZenFlex_NiTi_Rotary_Shaping_File_Brochure_MKT-20–0745_20201125_rev1_EN_US.pdf?u=18sth1 (date of access 19 May 2022)). Despite this, there are no data in literature regarding mechanical performance and metallurgical properties of those instruments.

According to this, the aim of this study was to evaluate the static mechanical properties (i.e., cyclic fatigue resistance, torsional resistance, bending ability) and the TTR of ZenFlex, comparing them with those of two comparable instrument brands: Vortex Blue (VB; Dentsply Tulsa Dental, Tulsa, OK, USA) and its replica-like system produced by EdgeEndo, the EdgeSequel Sapphire (ESS; EdgeEndo, Albuquerque, NM, USA).

## 2. Materials and Methods

The sample calculation was performed based on a previous study using G* Power v3.1 (Heinrich Heine, University of Düsseldorf, Düsseldorf, Germany) by setting an alpha-type error of 0.05, a beta power of 0.90, and an effect size of 0.80 [21]. According to this, 195 endodontic instruments were selected for the study: 65 25.04 ZenFlex (Group A), 65 25.04 Vortex Blue (Group B) and 65 25.04 EdgeSequel Sapphire (Group C). Before submitting the instruments to tests, a stereomicroscopic observation at 20× magnification was performed (Carl Zeiss Microimaging, Göttingen, Germany) with the purpose to evidence any manufacturing defects and none of them was found and then discarded.

According to the mechanical test and metallurgical analysis performed, the instruments of each group were divided in four subgroups as follow: subgroup 1 (*n* = 20) for the cyclic fatigue test, subgroup 2 (*n* = 20) for the bending test, subgroup 3 (*n* = 20) for the static torsional test and subgroup 4 (*n* = 5) for the differential scanning calorimetry (DSC) analysis. All mechanical tests were performed at ambient temperature (25 ± 1 °C).

For both cyclic fatigue and torsional tests, each instrument was rotated in a continuous motion at 500 rpm, with limit torque set respectively at 1.0 Ncm and 5.5 Ncm, using a 1:1 handpiece (Kavo, Biberach, Germany) connected to an electric motor (Kavo, Biberach, Germany).

### 2.1. Cyclic Fatigue Test

All the instruments of the three brands were rotated in a tapered stainless-steel artificial canal of 18 mm characterized by a 60° angle of curvature and a 5 mm radius of curvature, with a constant taper of 0.05 mm and a maximum diameter at 18 mm of 1 mm The artificial canal was wet with saline solution (NaCl 0.9%). Each instrument was inserted in the artificial canal until the first identification mark (indicating 18 mm length) reached the orifice of the canal.

After that, each instrument was freely rotated inside the artificial canal according to the manufacturers’ recommendation (500 rpm and torque limit set to 1.0 Ncm) until the fracture had occurred and the time from the activation of the instrument and the fracture was registered using a digital chronometer with a sensitivity of 0.01 s. Then, in order to determine the number of cycles to fracture (NCF), the following formula was adopted: NCF = revolutions per minute (rpm) × time to fracture (s)/60.

Moreover, the fractured fragments of each instrument were collected and measured with a digital caliper with a sensitivity of 0.01 millimeter in order to confirm the correct positioning of them during the cyclic fatigue test and to and to assess the quality of the methodology.

The length of the fragments (FL) and NCF of each group were statistically analyzed.

### 2.2. Bending Test

The bending test was performed using a custom-made device previously validated in recently published research [22]. 

All instruments were tested by the same operator in order to avoid any procedural error. The bending test was performed at a 45° angle and at 6 mm from the tip.

All measurements indicated by the load cell display were recorded, the mean values and the standard deviations were calculated and then, statistically analyzed. 

### 2.3. Static Torsional Test

The static torsional test was performed using an apparatus device consisting of: an 1:1 handpiece connected to an endodontic motor (Kavo, Biberach, Germany) able to record each 0.1 s the torque developed by the instrument with a sensitivity of 0.05 Ncm, and a vice used to firmly secure the instrument tip at 3 mm in a reproducible way. the static torsional test was performed blocking each instrument at 3 mm from the tip and rotating it at 500 rpm with the torque limit set to 5.5 Ncm until fracture had occurred. The torque to fracture (TtF) was registered by the dedicated motor.

Moreover, the fractured tips of each instrument were collected and measured with a digital caliper with a sensitivity of 0.01 mm. The FL was statistically analyzed in order to evaluate the correct insertion of the instruments tip imposed by the vice and to assess the quality of the static torsional test.

The mean values and the standard deviation of Fl and TtF of each group were calculated and statistically analyzed.

### 2.4. Differential Scanning Calorimetry (DSC)

The tests were carried out with a Perkin-Elmer DSC Pyris 8500 (Perkin-Elmer, Fremont, CA, USA) equipped with a cooling module. Purified nitrogen was the purge gas (30 mL min^−1^). The temperature and energy calibrations were performed using a pure indium standard and the baseline was obtained with an empty hermetically aluminum pan prior of each measurement. Segments of Vortex Blue, Sapphire and Zen Flex files (10 ± 1 mg), obtained by cutting the instruments, were weighed into aluminum pan and then hermetically sealed. An empty pan was used as reference. The samples were cooled at −70 °C, and then underwent to a heating/cooling cycle in the range −70 °C to 110 °C, with an isotherm step at 110 °C for one minute. The heating/cooling rate was 5 °C min^−1^. The tests were carried out in triplicate for each sample. Data processing was performed with PYRIS software.

The DSC measurement have been reported as mean values ± standard deviation and then statistically analyzed.

### 2.5. Scanning Electron Microscopy (SEM)

Scanning electron microscopies were performed to assess the causes of fracture arising from static mechanical tests (i.e., cyclic fatigue and torsional tests). The topographic features of the fractured surfaces were acquired observing five randomly selected fragments of NiTi rotary instruments of subgroups 1 and 3 under a high vaccum scanning electron microscope (VP-SEM; SU3550, Hitachi High Technologies Corporation, Tokyo, Japan). Each selected instrument was observed at two different magnifications (×250 and ×700) and acquired in secondary electrons imaging mode with the operating conditions set at an accelerating voltage of 8.00 kV and at a working distance between 4.8–6.6 mm.

### 2.6. Statistical Analysis

The length of the fragments (FL) arising from cyclic fatigue and torsional tests, NCF, bending force, TtF and DSC measurement of each group were statistically analyzed using a one-way analysis of variance (ANOVA) test with significance set to a 95% confidence level. Parametric tests were adopted after performing distribution normality test (Saphiro-Wilk test) with the significance level set to 0.05 using R software V 4.2.0. (R Core Team (2022), R Foundation for Statistical Computing, Vienna, Austria).

## 3. Results

All results derived from the mechanical tests are described below in the corresponding subparagraph and highlighted in Table 1.

### 3.1. Cyclic Fatigue Test

The results (mean values and standard deviation of time to fracture (s) and NCF) arising from the cyclic fatigue tests are shown in Table 1. Vortex Blue showed statistically significant higher values of time to fracture (s) and NCF than both ZenFlex and EdgeSequel Sapphire (*p* < 0.05), with NCF mean values and standard deviations respectively of 945.2 ± 111.6, 647.5 ± 92.5 and 626.7 ± 135.8, with the last two results not statistically significant (*p* > 0.05).

FL measurement showed a mean value and a standard deviation respectively of 5.86 ± 0.68 (mm) for VB, 6.17 ± 0.72 (mm) for ZF and 6.10 ± 0.41 (mm) for EES, with no statistically significant difference between them (*p* > 0.05).

### 3.2. Bending Test

The results (mean values and standard deviation of the force (g) generated to bend the instruments at 45° at 6 mm from the tip) arising from the bending tests are shown in Table 1. VB showed a statistically significant lower values of applied force (g) than both ZF and EES (*p* < 0.05), with mean values and standard deviations respectively of 55.3 ± 9.8 (g), 111.0 ± 12.9 (g) and 99.0 ± 11.4 (g), showing that EES and ZF have a higher stiffness and lower flexibility than VB.

### 3.3. Torsional Test

The results (mean values and standard deviation of TtF (Ncm)) arising from the torsional tests are shown in Table 1. VB and ZF showed the highest values of TtF (Ncm) with mean values and standard deviations respectively of 0.68 ± 0.13 (Ncm) and 0.59 ± 0.11 (Ncm), with no statistically significant difference between them (*p* > 0.05). On the contrary, EES showed the lowest values of TtF (0.40 ± 0.09 (Ncm)), showing the worst resistance to torsional stress in comparison to ZF and VB, with a statistically significant difference (*p* < 0.05).

FL measurement showed a mean value and a standard deviation respectively of 3.54 ± 0.42 (mm) for VB, 3.54 ± 0.52 (mm) for ZF and 3.61 ± 0.36 (mm) for EES, with no statistically significant difference between each subgroup (*p* > 0.05).

### 3.4. Differential Scanning Calorimetry (DSC) 

**Figure 1:**
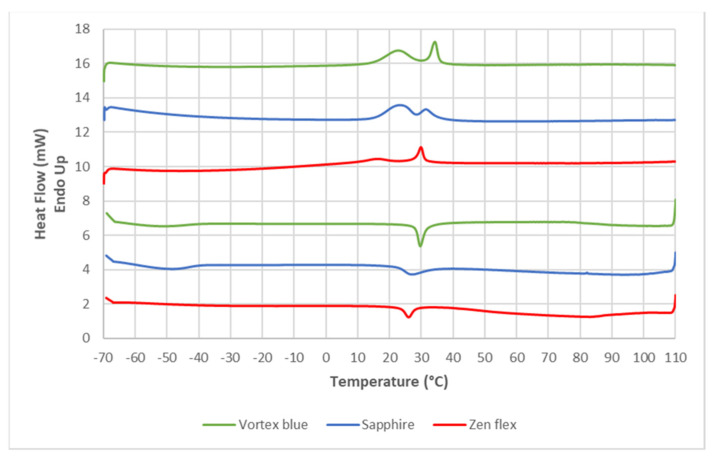
DSC curve of ZF, ESS and VB instruments with a heating/cooling rate of 5 °C. On the heating curves (top lines reading from left to right) two endothermic peaks were recorded for all three instruments, which showed the presence of R-Phase (first peak). On the contrary, a different thermal behavior was observed in the cooling curves (bottom lines reading from right to left), where the instruments showed only one relevant exothermic peak related to the reverse transformation of austenite to martensite. All data regarding TTR, peaks and ΔH are shown in Table 2.

The DSC heating and cooling curves of ZF, ESS and VB obtained from the analysis in aluminum pan were reported in Figure 1.

The DSC heating curves of the three tested instruments showed two endothermic peaks indicating the presence of R-phase. The R-phase is considered as an intermediate phase between the austenite and martensite, which occurs on cooling before the martensitic transformation is completed [20]. In the case of the ZenFlex instrument, the first thermodynamic event has a peak at a lower temperature (15.89 °C) than that of the Vortex Blue and Sapphire instruments (respectively 23.22 °C and 22.91 °C) and with an area that is about half that of the Vortex Blue and Sapphire instruments (30.91 mJ vs. 64.48 mJ and 56.82 mJ, respectively). Furthermore, the latter showed a similar As-Af temperature range and the Af is near the body temperature (36.15 °C and 34.40 °C, respectively), while for ZenFlex sample the transformation started and finished at lower temperatures (30.94 °C).

During the cooling phase, one exothermic event was recorded for the Vortex Blue, Sapphire and ZenFlex instruments (26.87 °C, 38.09 °C and 25.66 °C respectively).

The similar values of Area and ΔH calculated for the Vortex blue and Sapphire instruments in the heating phase, but higher than those of ZenFlex one, suggested that in those samples there was a more unstable martensite switching to the form of austenite than in the ZenFlex samples. The Transformation temperatures, Onset (As, Ms) and End (Af, Mf), and the related change in enthalpy (ΔH) are summarized in Table 2.

### 3.5. Scanning Electron Microscopy (SEM)

**Figure 2:**
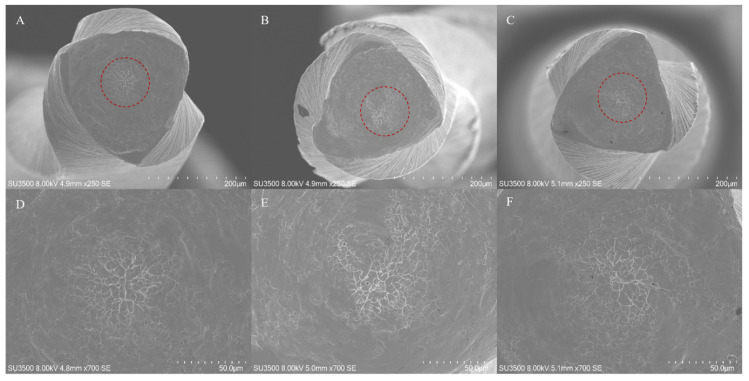
SEM micrographs of the fractured instruments after torsional tests. The typical fractographic pattern arising from excessive trosional load can observed in the micrographs (bottom line, ×700 magnification): central area of fibrous dimples near the center of rotation, surrounded by a peripherical zone of concentric circular abrasion marks. All SEM acquisition specifications are evidenced in each image. As shown in the images on the top (×250 magnification), all instruments are characterized by a trinagular cross-section. Upper line images: panoramic view of the fractured surfaces, (**A**) ZenFlex; (**B**) EdgeSequel Sapphire; (**C**) Vortex Blue. Bottom line images: particular of the upper images of the evidenced area (red dotted line) in which fibrous dimples near the center of rotation surrounded by concentric circular abrasion marks are evidenced at higher magnification, (**D**) ZenFlex; (**E**) EdgeSequel Sapphire; (**F**) Vortex Blue.

**Figure 3:**
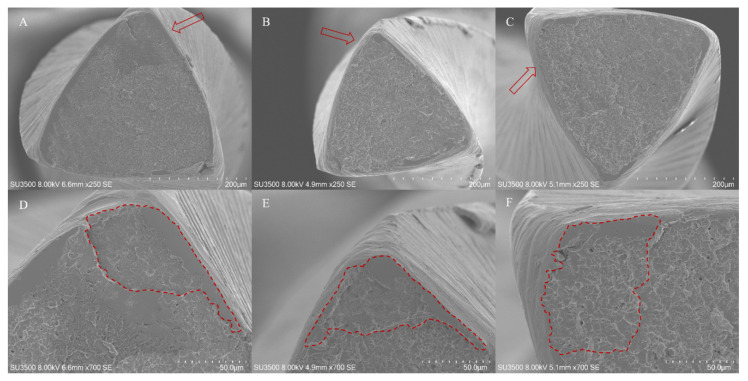
SEM micrographs of the fractured instruments after cyclic fatigue tests. The typical fractographic pattern arising from cyclic fatigue can observed in the micrographs (upper line, ×250 magnification): external origin of the cracks (red arrows) and a homogenous dimple area extended to the entire fractured surface. All SEM acquisition specifications are evidenced in each image. As shown in the images on the top (×250 magnification), all instruments are characterized by a triangular cross-section. Upper line images: panoramic view of the fractured surfaces, (**A**) ZenFlex; (**B**) EdgeSequel Sapphire; (**C**) Vortex Blue. Bottom line images: particular of the upper images of the evidenced area (red arrows) in which there are shown the areas of overload fast fracture zone with the external origin of the crack arising from cyclic fatigue, (**D**) ZenFlex; (**E**) EdgeSequel Sapphire; (**F**) Vortex Blue.

Fractographic pattern of separated instruments are shown in the Figure 2 and Figure 3. In the first one it is highlighted the typical feature of ductile fracture arising from cyclic fatigue failure characterized by an external origin of the cracks and a homogenous dimple area extended to the entire fractured surface. On the contrary, the fractured instruments arising from excessive torsional loads showed a central area of fibrous dimples near the center of rotation, surrounded by a peripherical zone of concentric circular abrasion marks. As evidenced in the micrographs, all instruments have a triangular cross-section.

## 4. Discussion

Considering the growing interest of companies on endodontics and the several new rotary systematics introduced into the market, their scientific validation should have a crucial role. This is fundamental to define how to clinically use them according to their mechanical and metallurgical properties, establishing their limits and merits. Taking into account the thorough interaction between mechanical behavior and metallurgy [1,20], as stated by Silva et al., the compartmentalization of knowledge should no longer be acceptable. A multimethod approach that takes into account both mechanical and metallurgical properties should be always preferred during the evaluation of endodontic rotary instruments in order to avoid a limited understanding of phenomena and in a gross oversimplification in knowledge application [23].

According to this, the aim of this study was to evaluate a recently introduced instrument (ZenFlex) through a static mechanical analysis and a differential scanning calorimetry, by comparing it with two well-known instruments that share with the former many similarities: the Vortex Blue and its replica-like systematic, the EdgeSequel Sapphire. As stated by manufacturer, the ZenFlex are characterized by a triangular cross-section with a 20% smaller maximum instrument diameter and a smaller mass in comparison to Vortex Blue, ensuring a higher cyclic fatigue resistance and torsional strength. Despite this, to date, there are no scientific data regarding ZenFlex, except for one article by Lin et al., in which the authors evaluated the vertical root fracture resistance of root canal-treated teeth instrumented with four different NiTi rotary file systems: HyFlex CM (Coltène/Whaledent, Altstätten, Switzerland), T-Pro and TG6 (Shenzhen Perfect Medical Instruments Co. Ltd., Guangdong, China), ZenFlex (Kerr Corporation, CA, USA) [24]. Nevertheless, no data are available both in term of mechanical properties and metallurgy of this instrument. Notwithstanding what affirmed by manufacturer, according to the results of this study, in terms of cyclic fatigue resistance and flexibility the ZF are comparable to ESS, but showed lower value of NCF in comparison to VB. Considering the reduced mass of the cross-section of ZF, this result was unexpected, since as stated by Grande et al. the lower is the mass the more is the cyclic fatigue resistance [19]. However, the explanation can be found in the DSC analysis which showed how ZF at ambient temperature is characterized by a more austenitic crystallographic phase than VB (Table 2). In fact, analyzing the TtF values, VB and ZF showed comparable results despite the above-mentioned difference in terms of mass, volume and thus polar moment of inertia (since the cross-sectional morphology is the same). The deficit in terms of mass/polar moment of inertia of ZF, which should decrease the torsional resistance [16], is balanced by an increased stiffness guaranteed by its slight proprietary heat-treatment. However, the assumption of the differences in terms of mass and cross-sectional volume should be considered as a hypothesis supported by the manufacturer since there are no studies which evaluated the instrument design. Moreover, it could be speculated that differences in terms of mechanical and metallurgical properties between the tested instruments could arise from differences in the elemental composition of the alloy. Since in this research the composition of the blank wires was not evaluated, further researches are needed to clarify this aspect.

Despite there are no data regarding ZF, there are two articles in literature that aimed to compare VB and ESS. Arias et al. evaluated the cyclic fatigue resistance of the VB and ESS systematics both at ambient (21 °C) and body temperature (37 °C) [21]. The authors found a statistically significant difference for tests performed at body temperature, with all VB instruments showing a higher cyclic fatigue resistance performed at 60° and 3 mm radius of curvature. Different results were found in case of ambient temperature, according to which small diameter (<0.25 mm) VB instruments were more resistant to fracture, whilst great diameter (>0.25 mm) ESS showed a higher cyclic fatigue resistance than VB. Regarding 25.04 instruments there was not found a statistically significant difference between two brands at ambient temperature (21 °C) [21]. These results do not corroborate those of our research since we found a statistically significant difference in terms of NCF between VB and ESS, with the former more resistant to fracture. The rationale behind those results can be found in the different condition of test. The most remarkable difference was the temperature at which the tests were performed. In our study the ambient temperature was set at 25 ± 1 °C, whilst Arias et al. set it at 21 °C [21]. Considering the increased gap between cyclic fatigue resistance at ambient and body temperatures observed by Arias et al., it could be speculated that the difference between the two studies in terms of temperature (about 5 °C) and, thus, of the crystallographic organization of the instruments is the reason of different results. Also Weyh et al. found the same results of our research in terms of cyclic fatigue resistance, corroborating our findings [25]. 

The DSC analysis of Arias et al., despite the different condition adopted during the calorimetry corroborates the result of our research, founding that VB and ESS would likely be in an austenitic state at body temperature, whilst the first is formed by R-Phase and martensite at ambient temperature while the latter is formed by martensite [21]. 

Moreover, despite the ESS manufacturer affirmed that those instruments are replica-like of VB, it cannot be excluded that there could be some differences in terms of design that could influence their mechanical properties. In fact, considering the result obtained by Alcade et al. in the evaluation of ProTaper Gold (Dentsply Sirona, Ballaigues, Switzerland) and its replica-like system by EdgeEndo (EdgeTaper Platinum; (Albuquerque, NM, USA)), some morphological differences were found [26]. However, there are no studies regarding this theme on VB and ESS, and further researches are needed to clarify it.

Despite there are no data regarding TTR and DSC analysis of ZF, it can be stated that at ambient temperature the instrument is characterized by R-Phase and martensite, whilst at body temperature it is formed by austenite. From DSC analysis it could be speculated that the proprietary heat-treatment by Kerr Corporation is softer than that of VB and ESS, since it showed a lower temperature of As and Af in comparison to the other instruments. On the contrary, the Ms and Mf temperatures of VB and ZF are almost comparable, whilst ESS showed higher temperature of Ms and Mf, highlighting that ESS at body temperature are in the Ms-Mf transformation (Ms = 43.71 ± 0.12 °C and Mf = 33.01 ± 0.04 °C).

The purpose of this research was to evaluate the mechanical performance of three comparable instruments, considering their difference in terms of heat-treatment and the related TTR. Despite this, their mechanical properties have been investigated through static tests, that, as stated by Silva et al., only partially represent the clinical scenario [23]. However, to date, static tests represent the best way allowing the variables isolation, increasing the validity and reproducibility of the study, making a first evaluation of endodontic systematics [23]. According to this, the main limitations of this research are fundamentally related to the absence of dynamic evaluation of the tested instruments, not considering important clinical characteristics such as shaping ability, cutting efficiency, dynamic cyclic fatigue resistance and dynamic torque-generated evaluation. For this reason, further researches are needed. 

## 5. Conclusions

According to the results of the study and considering its limitation, it can be concluded that VB showed the best mechanical performance during static tests in comparison to ESS and ZF. This is fundamentally due to the interaction of parameters such as instrument design and heat-treatments that are able to enhance its mechanical performance.

## Figures and Tables

**Table 1:** Schematic summary of the mechanical properties of ZenFlex, Vortex Blue and EdgeSequel Sapphire instruments resulting from cyclic fatigue, bending ability and torsional resistance. Different superscript letters in the same column indicate statistical differences among groups (*p* < 0.05).

	Cyclic Fatigue Test		Bending Test	Torsional Test
	Time (s)	NCF	Force (g)	TtF (Ncm)
ZenFlex	77.7 ± 11.1 ^a^	647.5 ± 92.5 ^a^	111.0 ± 12.9 ^a^	0.59 ± 0.11 ^a^
Vortex Blue	113.4 ± 13.4 ^b^	945.2 ± 111.6 ^b^	55.3 ± 9.8 ^b^	0.68 ± 0.13 ^a^
Sapphire	75.2 ± 16.3 ^a^	626.7 ± 135.8 ^a^	99.0 ± 11.4 ^a^	0.40 ± 0.09 ^b^

**Table 2:** Phase transformation temperatures and associated energy of VB, ESS and ZF (mean ± SD, *n* = 3), tested in aluminum pans.

	Heating Process				
	A_s_(Onset) (°C)	Peak (°C)	A_f_ (end) (°C)	Area (mJ)	ΔH (J/g)
Vortex blue peak 1	17.07 ± 0.92	23.22 ± 0.48	28.36 ± 0.64	64.48 ± 2.18	5.35 ± 0.18
Vortex Blue peak 2	32.65 ± 0.42	35.26 ± 1.34	36.15 ± 0.62	34.69 ± 2.47	2.89 ± 0.21
Sapphire peak 1	17.77 ± 0.64	22.91 ± 0.03	27.16 ± 0.04	56.82 ± 2.11	1.42 ± 0.13
Sapphire peak 2	29.04 ± 0.36	31.27 ± 0.15	34.40 ± 0.37	22.51 ± 1.06	4.74 ± 0.18
Zen flex peak 1	11.80 ± 0.73	15.89 ± 0.57	19.64 ± 0.31	30.91 ± 0.09	3.09 ± 0.01
Zen flex peak 2	28.13 ± 0.12	29.74 ± 0.25	30.94 ± 0.33	20.43 ± 0.50	2.04 ± 0.60
	Cooling Process				
	M_s_ (Onset) (°C)	Peak (°C)	M_f_ (end) (°C)	Area (mJ)	ΔH (J/g)
Vortex Blue	31.36 ± 0.22	26.87 ± 0.06	23.30 ± 0.04	−41.88 ± 1.05	−3.86 ± 0.19
Sapphire	43.71 ± 0.12	38.09 ± 0.02	33.01 ± 0.04	−23.65 ± 0.73	−2.26 ± 0.22
ZenFlex	27.89 ± 0.04	25.66 ± 0.92	23.99 ± 0.16	−25.49 ± 2.45	−2.55 ± 0.24
